# Feasible Nipple Preservation Techniques for Breast Cancer With Suspected Ductal Spread to the Nipple Base on Imaging: A Case Report

**DOI:** 10.7759/cureus.105282

**Published:** 2026-03-15

**Authors:** Mami Yoshida, Shoji Oura

**Affiliations:** 1 Department of Surgery, Kishiwada Tokushukai Hospital, Kishiwada, JPN

**Keywords:** breast cancer, ductal spread, nipple base, nipple necrosis, nipple preservation techniques

## Abstract

Possible ductal spread to the nipple base on imaging generally leads breast surgeons to perform either partial mastectomy, including nipple resection, or total mastectomy in breast cancer patients. Our nipple base resection techniques, however, can provide safe nipple preservation for many breast cancer patients, even when imaging suggests ductal spread to the nipple base. An 86-year-old woman with breast cancer near the nipple-areolar complex showed suspected ductal spread to the nipple base on preoperative imaging. Due to the patient’s strong preference for nipple preservation, she underwent nipple preservation using our techniques as follows. A spindle skin resection was performed just above the breast cancer and connected to the peri-areolar incision, followed by skin flap formation in a thick-flap manner, extending to areas sufficient for partial mastectomy. A partial mastectomy was performed with an incision of the mammary gland distal to the tumor, blunt dissection of the retro-mammary space around the breast cancer using fingers, further incision of the mammary gland toward the nipple under palpation of the breast cancer to avoid an off-center partial mastectomy, and complete skeletonization of the sub-nipple mammary gland. Thereafter, the nipple base was resected with scissors while the nipple was slightly depressed by pulling the partial mastectomy tissue. These procedures led to quasi-complete resection of the target mammary gland, showed pathologically negative margins at the nipple base, and caused no complications to the nipple-areolar complex. The patient has been well for six months on endocrine therapy without adjuvant radiotherapy due to both her advanced age and her preference. Breast surgeons should note that our nipple preservation techniques can safely preserve the nipple even in cases of breast cancer with suspected ductal spread to the nipple base on imaging.

## Introduction

Breast-conserving therapy has provided significant benefits to many breast cancer patients worldwide for more than three decades [1,2]. In addition to tumor size, it has several exclusion criteria, such as multicentricity and tumor location [3,4]. Breast surgeons, however, have long recognized that ductal spread is the most important exclusion factor for breast-conserving therapy [5,6].

Improvements in ultrasound image resolution and the ability to more precisely predict multicentricity and ductal spread in the ipsilateral breast using MRI have enabled breast surgeons to perform partial mastectomies more accurately [7]. For example, an accurate partial mastectomy may require the resection of the overlying skin when imaging shows cancer cell infiltration toward the skin and performing a partial mastectomy in a sector rather than a round fashion when there is suspected ductal spread toward the nipple. However, breast surgeons very often choose to operate on breast cancers with possible ductal spread up to the nipple base, not with sector resection, but with either partial mastectomy, including nipple resection, or total mastectomy. Conversely, surgeons often encounter situations in which preoperative imaging suggests ductal spread to the nipple base, but pathological evaluation reveals no cancer infiltration at the nipple base.

If feasible nipple-preserving techniques that safely and completely remove the sub-nipple mammary gland were available, nipple preservation would be possible for many breast cancer patients in whom preoperative imaging suggests ductal spread to the nipple base. We have over 30 years of experience in nipple-sparing surgery and have explored methods to safely preserve the nipple [8]. Therefore, we have developed a feasible method for excising ductal spread extending to the nipple base.

We herein report our nipple preservation techniques by demonstrating actual surgical procedures for breast cancer with suspected ductal spread to the nipple base on imaging, resulting in successful nipple preservation with pathologically proven negative surgical margins at the nipple base.

## Case presentation

**Figure 1:**
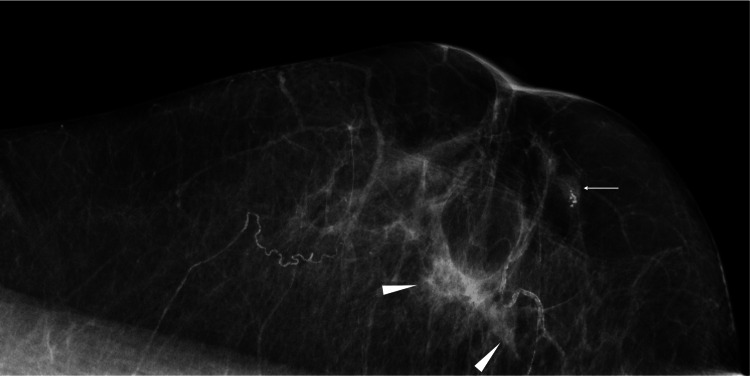
Mammography findings Mediolateral view mammography showed an indistinct mass (arrowheads) and a presumed daughter nodule (arrow).

An 86-year-old woman visited our hospital, Kishiwada Tokushukai Hospital, Kishiwada, Japan, after noticing a mass in the upper-inner quadrant of her right breast. The patient had a history of depression, a family history of colon cancer in her daughter, and no physical abnormalities except for the breast mass. Mammography showed an obscured mass and a presumed daughter nodule in the direction of the nipple (Figure 1).

**Figure 2:**
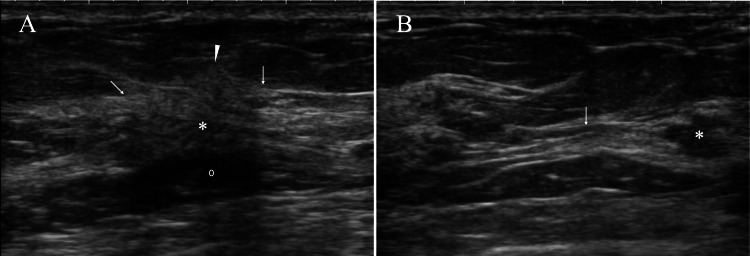
Ultrasound findings (A) Ultrasound showed an indistinct mass (asterisk), disruption of the anterior borders of the mammary gland (arrows), hyperechoic areas just above the mammary gland (arrowhead), and slightly attenuated posterior echoes (open circle). (B) Ultrasound showed a daughter nodule (asterisk) in the nipple direction of the main tumor and tubular low echoes (arrow) extending from the daughter nodule toward the nipple.

Ultrasound revealed disruption of the anterior borders of the mammary gland, a hyperechoic pattern with indistinct margins (so-called halos) just above the mammary gland, a presumed daughter nodule, and duct dilatation extending from the daughter nodule toward the nipple (Figure 2).

**Figure 3:**
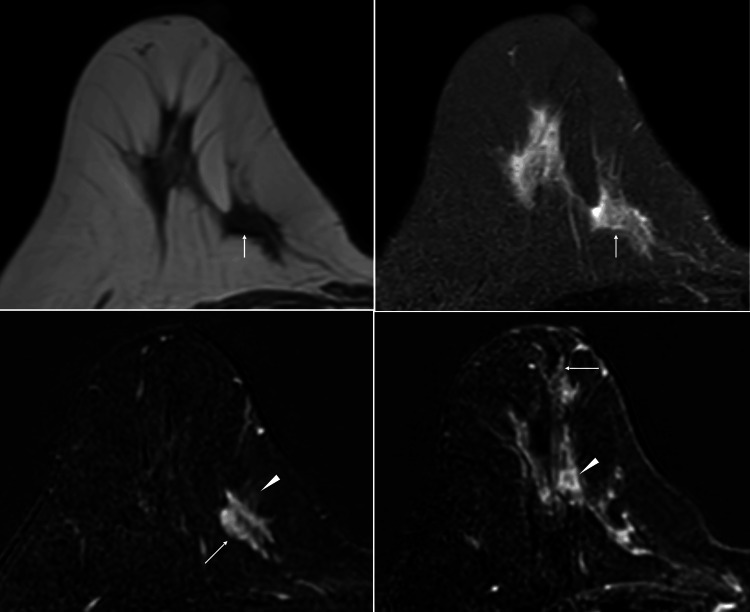
MRI findings MRI of the tumor showed low signals on T1-weighted images (A, arrow), slightly high signals on fat-suppressed T2-weighted images (B, arrow), fast enhancement (C, arrow), enhancement of the cancer cell infiltration areas in the subcutaneous fat (C, arrowhead), and a daughter nodule (D, arrowhead) with presumed ductal spread (D, arrow) on dynamic studies.

MRI demonstrated that the tumor had low signals on T1-weighted images, weakly high signals on T2-weighted images, a slow/persistent enhancement pattern on dynamic studies, and a suspected daughter nodule with linear enhancement extending up to the nipple base (Figure 3).

Core needle biopsy pathologically showed atypical cells growing in linear and cord-like patterns with stromal connective tissue proliferation, leading to a diagnosis of scirrhous-type invasive ductal carcinoma. Immunostaining revealed estrogen and progesterone receptor positivity (both Allred scores of 8), human epidermal growth factor receptor type 2 negativity, and a Ki-67 labeling index of 14%. The patient’s strong preference for nipple preservation led us to attempt breast-conserving surgery with nipple preservation, accompanied by sentinel node biopsy.

During the operation, two sentinel nodes were identified through a small skin incision in the axilla and confirmed negative for metastasis on frozen section. Details of our nipple preservation techniques were as follows. We began the operation with a spindle-shaped skin resection just above the breast cancer, including the core needle biopsy puncture site, and connected it to the peri-areolar skin incision to facilitate resection of the presumed ductal spread toward the nipple base. Skin flaps were then elevated in a thick-flap manner (i.e., flap formation between the subcutaneous fat and the mammary gland) except in the immediate vicinity of the breast cancer. Partial mastectomy was initiated by incising the mammary gland distal to the breast cancer with 1.5-cm safety margins. After reaching the retro-mammary space, we bluntly dissected it with fingers and continued the partial mastectomy while palpating the main tumor and the daughter nodule to avoid off-center resection, extending toward the nipple base.

**Figure 4:**
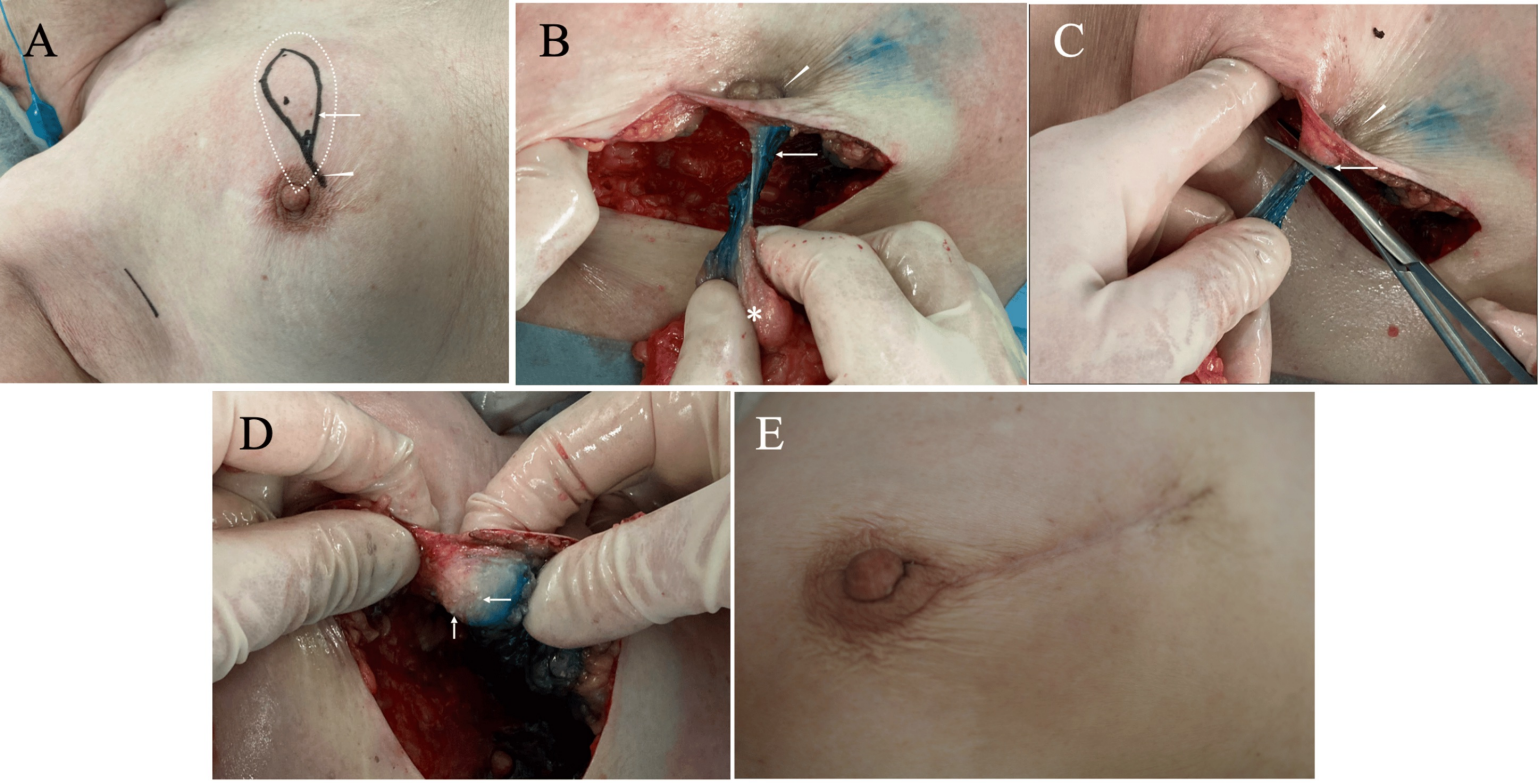
Operative procedures (A) Spindle skin resection was performed just above the breast cancer, including the core needle biopsy puncture site (arrow), and connected to the peri-areolar skin incision (arrowhead). The white dotted teardrop-like shape illustrates the partial mastectomy areas performed in a sector fashion under the skin. (B) Before nipple base resection, the sub-nipple mammary gland was completely skeletonized (arrow), with fat tissue around it removed, and the degree of nipple depression was checked by pulling the partial mastectomy tissue (arrowhead, asterisk). (C) During nipple base resection, quasi-complete resection of the mammary gland in and just beneath the nipple was performed to keep the nipple slightly depressed (arrowhead) and avoid damage to the nipple envelope base (arrow). (D) The reversed nipple, pushed outward with a finger, showed several stumps of the intra-nipple mammary ducts (arrows), indicating complete resection of the sub-nipple mammary gland and quasi-total resection of intra-nipple ducts. (E) The preserved nipple showed no complications four weeks after surgery.

After fully skeletonizing the sub-nipple mammary gland, we ultimately resected it using scissors while the nipple was slightly depressed from the surrounding skin by pulling the partial mastectomy specimen. This approach resulted in macroscopic quasi-complete mammary gland resection and the pathologically confirmed absence of cancer cells at the nipple base on frozen section (Figure 4A-4D).

**Figure 5:**
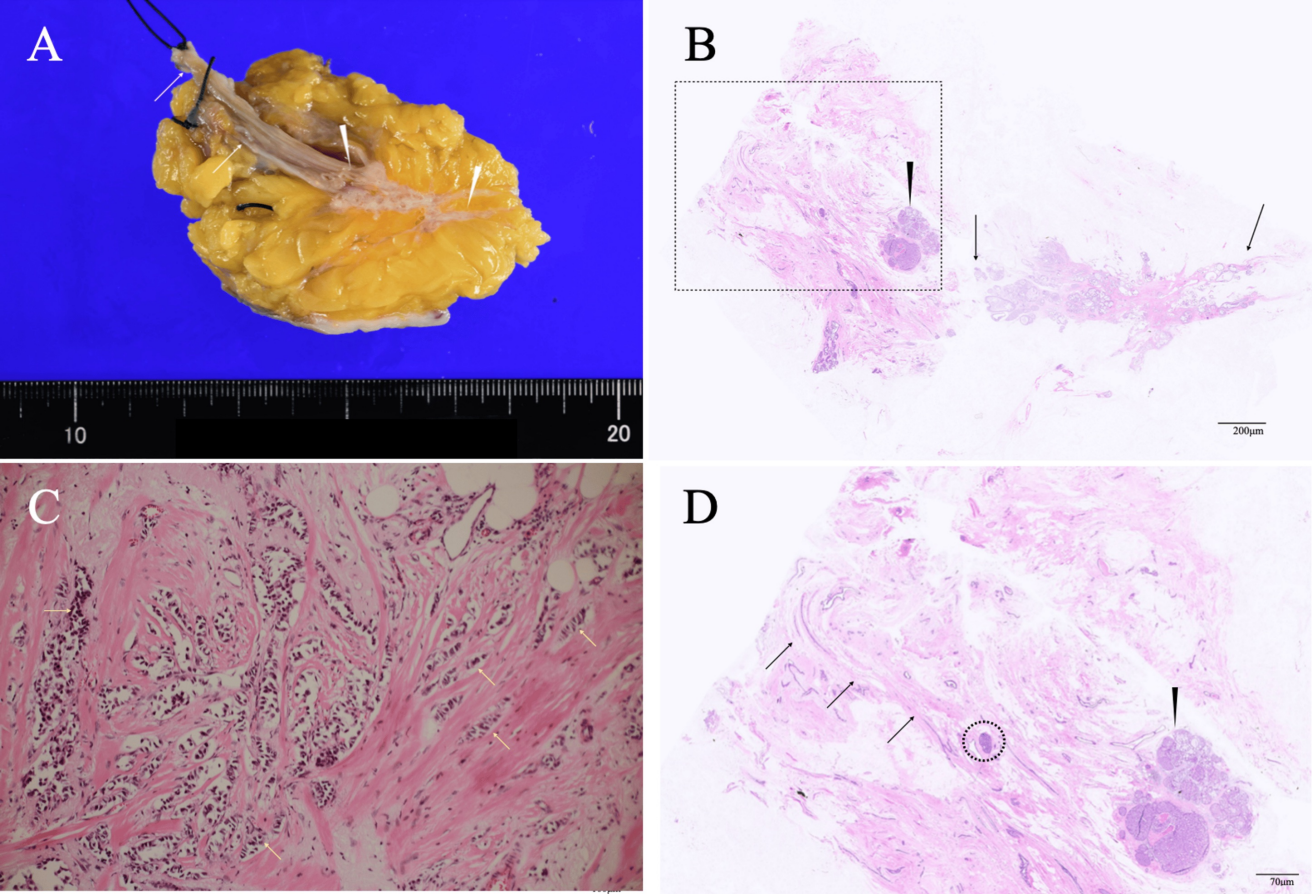
Pathological findings (A) Bisected tumor showed an indistinct mass (arrowheads) and macroscopically intact mammary gland (arrows) just beneath the nipple. (B) Low-magnification view showed the indistinct mass (arrows) and a daughter nodule (arrowhead). (C) In addition to the predominant distribution of noninvasive ductal cancer foci, the magnified view showed invasive cancer cells (arrows) sparsely spread throughout the tumor. (D) Magnified view of the dotted square in panel B showed a daughter nodule (arrowhead), a very small noninvasive cancer focus (dotted open circle), and no cancer spread near the nipple base (arrows).

Finally, the tissue defect caused by partial mastectomy was filled through breast tissue mobilization without nipple resection after confirming negative margins at the nipple base on frozen section. Postoperative pathological examination showed the main tumor consisting of cancer cells growing in linear, cord-like, cribriform, and tubular patterns; the daughter nodule consisting of noninvasive atypical cells in a cribriform pattern; and no intraductal cancer foci at the mammary duct stumps in the nipple (Figure 5).

The patient recovered uneventfully, was discharged on the second postoperative day, showed no complications in the nipple-areolar complex (Figure 4E), began receiving an aromatase inhibitor as adjuvant therapy without radiotherapy to the breast due to her preference, and has been well for six months.

## Discussion

Markedly improved resolution of ultrasound images allows us to perform more delicate breast-conserving surgery on a case-by-case basis rather than relying on traditional partial mastectomies with pre-set safety margins [9]. In this case, ultrasound images strongly suggested cancer cell infiltration into the fat due to the presence of hyperechoic areas in the subcutaneous fat, prompting complete resection of the skin and subcutaneous fat just above the main breast cancer.

When determining indications for breast-conserving therapy, it is crucial for diagnostic physicians to evaluate multicentricity and ductal spread of breast cancer using MRI. Ultrasound can also evaluate daughter nodules and ductal spread to some extent [10], but MRI is far superior, especially for detecting ductal spread. In this case, MRI more clearly depicted cancer cell distribution in the breast than ultrasound.

Breast surgeons generally have three options when detecting suspected ductal spread to the nipple base on imaging. The first option is mastectomy, which provides the best local control for breast cancer patients. The second option is breast-conserving surgery with concurrent nipple resection, followed by delayed nipple reconstruction if requested. The third option, used in this case, is partial mastectomy with nipple preservation by maximal resection of ductal spread, which can be feasible if pathologically negative surgical margins at the nipple base are confirmed [11,12]. In this case, our nipple base resection techniques caused no complications to the nipple and pathologically showed negative margins at the nipple base, despite suspected ductal spread on both MRI and ultrasound.

The vast majority of breast surgeons are primarily concerned about nipple necrosis when performing nipple base resection. However, few surgeons recognize that nipple congestion, generally caused by damage to the drainage veins of the nipple, rather than ischemia, plays the most important role in the development of nipple necrosis [13,14]. Therefore, we preserve the nipple in cases of presumed ductal spread to the nipple base by creating thick skin flaps, especially around the nipple-areolar complex, followed by complete skeletonization of the sub-nipple mammary gland. We clarify the nipple base by pulling the partial mastectomy tissue and leaving minimal mammary gland tissue in the nipple. In this case, the stumps of intra-nipple mammary ducts could be confirmed macroscopically, strongly suggesting quasi-complete resection of the mammary gland beneath and within the nipple.

We had planned to core out the nipple if surgical margins within the nipple were positive on frozen section, ensuring ultimate negative margins even in breast cancer with ductal spread up to the nipple apex. This patient did not undergo adjuvant radiotherapy to the conserved breast due to a strong preference. Our nipple base resection techniques, therefore, can provide safer nipple preservation in patients receiving adjuvant radiotherapy after nipple preservation surgery.

This study has several limitations, including favorable clinical outcomes in only a single case, a very short postoperative follow-up period, and no adjuvant radiotherapy to the conserved breast. Future studies are needed to determine whether similar favorable results can be achieved using our nipple base resection procedures in a larger cohort with longer follow-up periods.

Preoperatively, we speculated that this breast cancer had favorable biology with low proliferative potential based on pathological and immunostaining findings from the core needle biopsy specimen. Imaging also strongly suggested cancer cell spread toward the overlying skin. Surgeons should pay careful attention to the thickness of preserved subcutaneous fat around the main tumor during breast-conserving surgery. We believe that breast cancer with suspected ductal spread to the nipple base on imaging can be safely treated with our nipple preservation techniques.

## Conclusions

Accurate evaluation of multicentric foci and ductal spread is essential for diagnostic physicians in selecting appropriate surgical options for breast cancer. Breast surgeons should note that nipple preservation with pathologically confirmed negative margins at the nipple base can be a viable alternative to surgical options that include nipple resection. In addition, surgeons should be aware of techniques to safely and maximally resect ductal spread around the nipple.
